# Monitoring of respiration and cardiorespiratory interactions from multichannel seismocardiography signals

**DOI:** 10.1007/s13246-025-01657-4

**Published:** 2025-10-06

**Authors:** Jessica Centracchio, Salvatore Parlato, Samuel E. Schmidt, Paolo Bifulco, Daniele Esposito, Emilio Andreozzi

**Affiliations:** 1 Department of Electrical Engineering and Information Technologies, University of Naples Federico II, Via Claudio, 21, 80125 Naples, Italy; 2 Department of Health Science and Technology, Aalborg University, Selma Lagerløfs Vej 249, 9260 Gistrup, Aalborg, Denmark; 3 Department of Information and Electrical Engineering and Applied Mathematics, University of Salerno, Via Giovanni Paolo II, 132, 84084 Fisciano, Italy

**Keywords:** Accelerometer, Cardiorespiratory interactions, Cardiac monitoring, Respiration, Seismocardiography

## Abstract

Seismocardiography (SCG) uses accelerometers to record cardiac-induced accelerations of the chest wall. Cardiorespiratory interactions cause changes in amplitude and morphology of the SCG signals. Accelerometers can also directly monitor respiration by tracking thoracic inclination. This study thoroughly investigated the influence of accelerometer placement on the monitoring accuracy of respiration and cardiorespiratory interactions from SCG signals. Simultaneous recordings acquired by 16 accelerometers and a respiration belt placed onto 9 subjects’ chests were analyzed. Respiratory signals were estimated considering: (a) chest inclination, (b) amplitude modulation (AM) and (c) morphological changes of SCG signals for each sensor location. For the first time in literature, a continuous description of respiratory-induced changes in SCG morphology was obtained via a morphological similarity index (MSi). The performance of respiratory acts detection and inter-breath intervals (IBIs) estimation was evaluated against the concurrent reference respiration signal. High accuracy was achieved in all three kinds of respiratory signals, with average sensitivity and positive predictive value of 95.8% and 95.5% for chest inclination, 85.9% and 84.4% for AM, 94.3% and 95.7% for MSi. Moreover, IBIs measurements showed non-significant biases and limits of agreement of about ± 0.8 s for chest inclination and MSi, and ± 1 s for AM. Performance achieved by chest inclination and MSi appeared not much influenced by sensor location, while AM showed higher variations. Information on breathing and cardiorespiratory interactions can be accurately obtained via SCG on multiple sites on the chest.

## Introduction

Cardiomechanical monitoring techniques record small body vibrations generated by the mechanical activity of the cardiovascular system. These techniques use different mechanical sensors applied on various body sites to provide important insights into cardiovascular mechanical activity. Ballistocardiography (BCG) [1, 2] records the recoil forces of the body in reaction to blood ejection into the vasculature by means of ad hoc weighing scales, force or pressure sensors. Phonocardiography (PCG) [3, 4] captures the acoustic vibrations of the chest wall, commonly known as heart sounds, by microphones. Seismocardiography (SCG) [5–9] measures the precordium accelerations via lightweight accelerometers, manufactured via micro-electromechanical systems (MEMS) technologies. Similarly to SCG, Gyrocardiography (GCG) [10, 11] measures the angular velocities of the precordium via MEMS gyroscopes. Forcecardiography (FCG) [12–15] uses broadband force sensors to monitor respiration, ventricular volume variations, infrasonic cardiac vibrations, and heart sounds, all simultaneously from a single contact point on the chest. Particularly, the infrasonic FCG components share very high similarity to the accelerometric SCG signals, and therefore allow accurate localization of aortic valve opening and closure events and estimation of pre-ejection period (PEP) and left ventricular ejection time (LVET) [13, 15].

Cardiomechanical signals are known to be affected by respiration due to cardiorespiratory interactions [16–19]. Indeed, the heart is often referred to as “*a pressure chamber within a pressure chamber*” because it is surrounded by the lungs [20, 21]. Therefore, heart and lungs apply variable pressures on each other, both via direct physical contact and through the blood vessels they are connected with [20–22]. Changes in intrathoracic pressure during respiration cause alterations in ventricular preload, afterload, and contractility, which in turn affect the pressure gradients across the heart valves [23, 24], and, eventually, the onset, duration, and force of ventricular contractions [25]. Various parameters of cardiac function are influenced by these cardiorespiratory interactions, e.g., heart rate, amplitude and timings of heart valves movements, and stroke volume [26–28]. Ultimately, cardiorespiratory interactions reflect on cardiomechanical signals as modulation of various features, mainly amplitude and time distances between specific fiducial points. Moreover, most of the sensors used by cardiomechanical monitoring techniques are also capable of capturing respiratory movements of the chest, thus providing additional, purely respiratory signals, which do not carry any information about cardiac activity. Various studies have described the use of accelerometers to record changes in chest inclination during the breathing activity [29–33] and also the typical cardiac signals referred to as seismocardiograms, which are affected by cardiorespiratory interactions as well [9, 16–19, 26–28, 34, 35]. Azad et al. extracted three respiratory signals from SCG recordings, namely baseline wander (i.e., chest inclination), amplitude modulation, and inter-beat interval modulation (i.e., respiratory sinus arrhythmia) [17]. Pandia et al. analyzed the respiratory-induced modulation of inter-beat interval, a surrogate measure of LVET, and the amplitude ratio of systolic and diastolic complexes [16]. Differently from these studies, Taebi and Mansy focused on changes in SCG signals morphology that occur during respiration [18]. They investigated the similarity of SCG heartbeats belonging to the same respiratory phase (i.e., inspiration or expiration) or to the same level of lung volume (i.e., low volume or high volume). Particularly, lung volume turned out to be the main cause of respiratory-induced changes in SCG morphology. However, the methodology proposed by Taebi and Mansy provided a discrete description of these morphological changes by considering only two SCG heartbeat waveforms. Recently, Dhar et al. investigated morphological variations in SCG signals due to respiration by extracting different features and grouping them into eight respiratory phases. Similarly to previous studies, they confirmed that both temporal features, such as the inter-beat interval, PEP, LVET, and diastolic time, and amplitude features, such as the amplitude of diastolic complex, change with respiration [35].

A more in-depth investigation of the morphological changes in cardiomechanical signals due to cardiorespiratory interactions was carried out within the scope of FCG [15]. Particularly, an index of morphological similarity between single heartbeats, namely MSi, was extracted from the high-frequency component of FCG signals to obtain a continuous description of the beat-by-beat changes in FCG heartbeat morphology caused by respiration. Moreover, in the same study, the respiratory-induced modulation of LVET was analyzed. Both the MSi and the LVET have been found to exhibit cyclic time trends that highly resemble reference respiratory signals. These two respiratory-modulated parameters demonstrated high consistency within the respiratory cycle, thus providing accurate localization of respiratory acts and estimation of inter-breath intervals (IBIs). Furthermore, in another FCG study [14], respiration has been found to cause amplitude modulation of both low-frequency and high-frequency FCG components. Finally, FCG sensors have been proven capable of recording purely respiratory signals by capturing chest wall movements during respiration. Indeed, a very low-frequency component, like a large baseline wander, can be extracted from the raw FCG signals that reflects the forces impressed by the chest expansions and releases during the breathing acts [12].

This study investigated the impact of SCG sensor placement on the extraction of respiratory information from multichannel SCG signals from a public database [36]. Particularly, chest inclination, as well as amplitude modulation and beat-to-beat morphological variation of SCG signals were evaluated.

The novelty and main contribution of this study are three-fold:

1. For the first time, a continuous description of morphological changes in SCG signals is presented, as opposed to previous studies that proposed discrete descriptions based only on two SCG morphologies;
2. A thorough evaluation of the relationship between respiration and the related alterations induced on SCG signals is carried out;
3. The impact of SCG sensor placement on the extraction of respiratory information is addressed for the first time.

## Methods

### Dataset

**Fig. 1:**
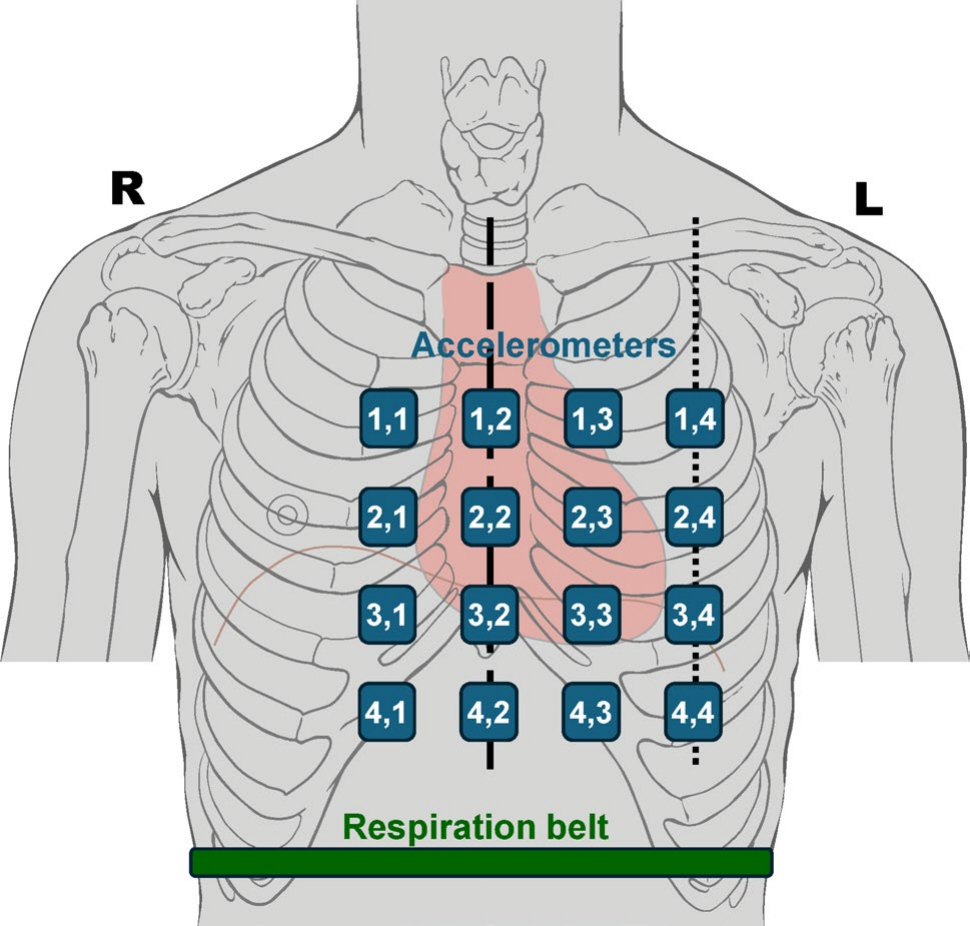
Positioning of the 4-by-4 accelerometer matrix and the respiration belt on a subject’s chest

Multichannel SCG signals from a publicly available database [36] were analyzed in this study. The database consists of simultaneous SCG recordings acquired via a 4-by-4 matrix of triaxial accelerometers from 13 male subjects (age 27 ± 4 years, BMI 24 ± 4 kg/m^2^), together with 3-lead ECG and a respiration belt signal [37]. Particularly, as described in [37], the multichannel SCG signals were acquired using 16 ADXL355 triaxial accelerometers (Analog Devices, USA) characterized by 3.9 µg/bit resolution, 25 µg/√Hz noise level and linear frequency response in the 0–1000 Hz frequency range. The accelerometers were housed into 3D-printed plastic casings of 30 mm × 30 mm × 18 mm size, connected by ribbon cables, and assembled in a 4-by-4 matrix at approximately 40 mm center-to-center distance, thus resulting in a 9 cm^2^ sensors area. The accelerometers were supplied with a synchronization signal by an ADS1298® analog front-end (Texas Instruments, USA). The ADS1298 was used to acquire a 3-lead ECG and a respiration belt signal. The accelerometers and the ADS1298 front-end were connected to one of the three FT51® microcontrollers (FTDI Chip, USA) located on the motherboard. The microcontrollers were connected to a computer via USB and transferred data using the SPI communication protocol implemented with the LTC6820 interface (Linear Technology, USA). The 4-by-4 matrix was placed onto the subject’s chest, together with four ECG electrodes, and the respiration belt was mounted around the abdomen, as depicted in Fig. 1. The 4 columns of 4 accelerometers were placed along the right parasternal line, the midsternal line, the left parasternal line, and the midclavicular line, so that the accelerometer in the location (3,2) was placed at the xiphoid process. 16-channel SCG, ECG and respiration signals were simultaneously acquired at 500 Hz sampling rate while the subjects were asked to lie in a supine position under quite breathing for about 3 min. After a visual inspection, the multichannel SCG signals of four subjects (i.e., subjects #8, 9, 10 and 13) were excluded from the analysis because of poor signals quality. Therefore, the signals of 9 subjects were analyzed.

### Signals pre-processing

All the processing operations described in this study were performed in MATLAB® R2023b (MathWorks, Inc., Natick, MA, USA). As a first step, the cranio-caudal y-axis SCG (y-SCG), the dorso-ventral z-axis SCG (z-SCG) and the respiration belt signals were linearly interpolated at 1 kHz via the MATLAB® function “*interp1*” to improve their temporal resolution. Afterwards, the interpolated signals were high-pass filtered using a zero-lag 2nd order Butterworth filter with 0.05 Hz cut-off frequency to remove their DC components. Finally, the respiration belt signal was low-pass filtered via a zero-lag 4th order Butterworth filter with 0.5 Hz cut-off frequency to filter out spurious oscillations at higher frequencies. After signal pre-processing, distinct procedures were carried out to pursue three main objectives: (1) to extract a respiration-related component from the baseline oscillation of the y-SCG signals; (2) to extract the respiratory-induced amplitude modulation of the z-SCG signals; (3) to extract the respiratory-induced morphology variation of the z-SCG signals.

### Extraction of respiration from y-SCG signals

The expansions and relaxations of the thorax during breathing activity cause changes in the inclination of the accelerometer matrix. In the supine position, the y-axis acceleration is the most sensitive for measuring chest motion with respiration, as it reflects the variation in the acceleration of gravity measured along the y-axis [33, 34]. Therefore, the y-SCG signals were processed by extracting a very low-frequency component related to respiration via a 3rd order Savitzki-Golay smoothing filter [38] with a frame length of about 3 s. The respiratory signals thus obtained, referred to as R-SCG, were further low-pass filtered via a zero-lag 4th order Butterworth filter with 0.5 Hz cut-off frequency.

### Extraction of amplitude modulation of z-SCG signals

**Fig. 2:**
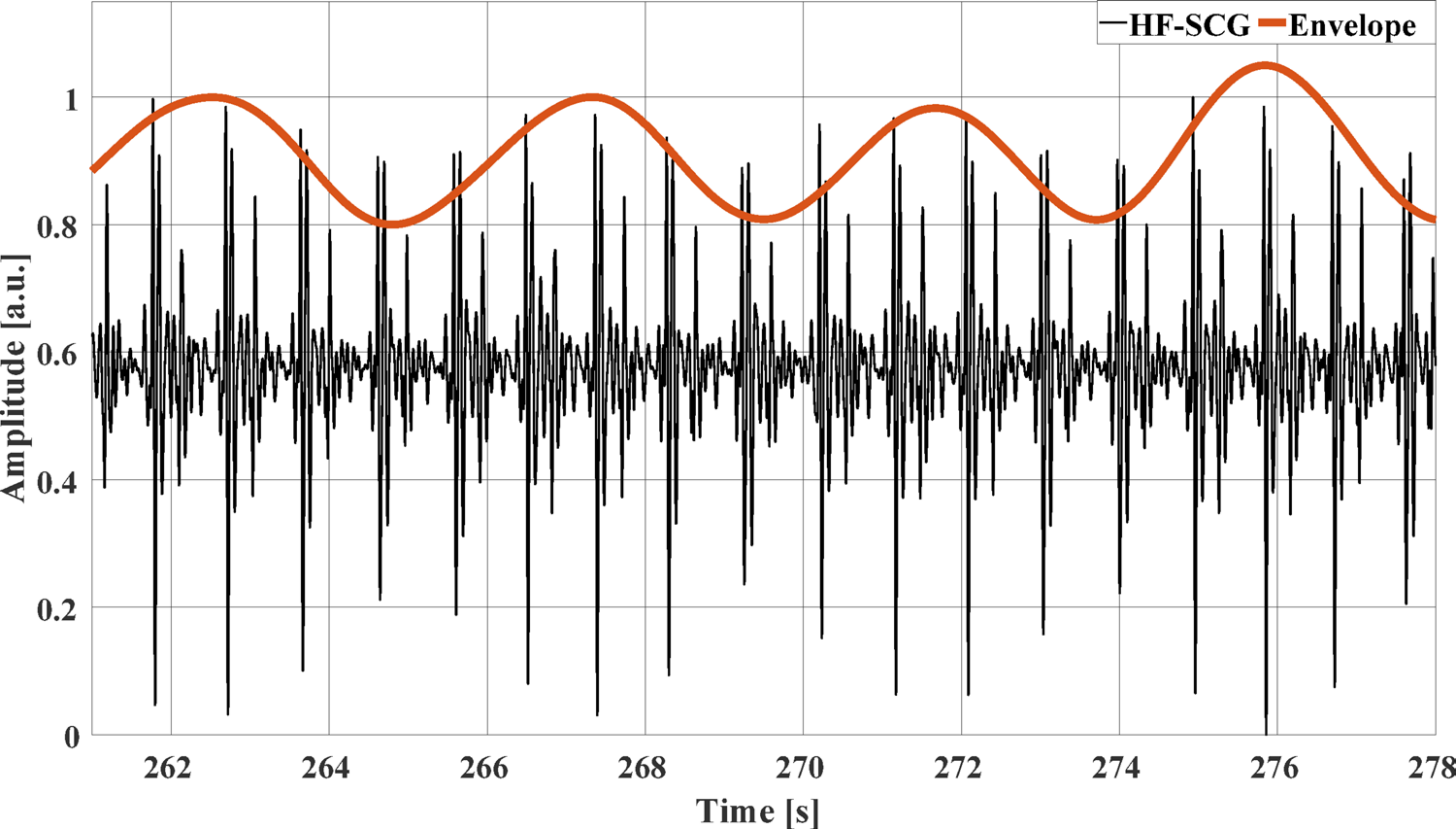
An excerpt of the HF-SCG signal (black line) and its envelope (red line) from subject #4 at the location (3,3)

The z-SCG signals were processed to isolate their cardiac components. As in [39, 40], the respiratory component was first extracted via a 3rd order Savitzki-Golay filter [38] with a frame length of about 3 s. Then, the z-SCG signals were deprived of their respiratory components and band-pass filtered via a zero-lag 4th order Butterworth filter with cut-off frequencies of 7 and 30 Hz to obtain the HF-SCG signals. As in [14], to obtain information on the amplitude modulation, an envelope was extracted from the HF-SCG signals by performing square rectification followed by low-pass filtering via a zero-lag 4th order Butterworth filter with 0.5 Hz cut-off frequency. Figure 2 shows an excerpt of the HF-SCG signal acquired from subject #4 at the location (3,3) and the envelope extracted from it.

### Extraction of morphology variation of z-SCG signals

**Fig. 3:**
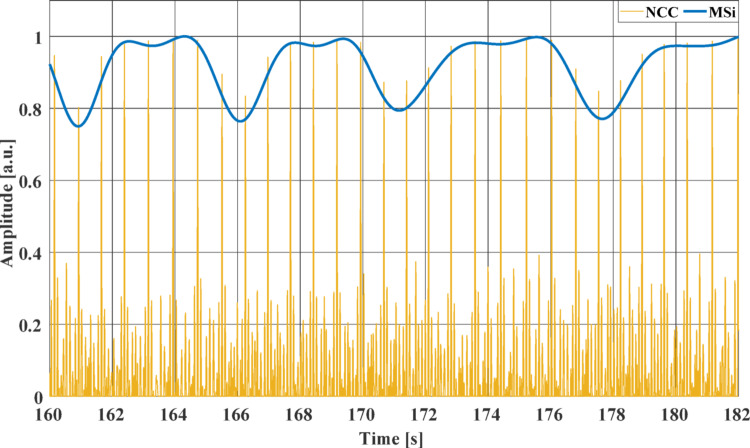
An excerpt of the NCC signal (yellow line) and the MSi signal (blue line) from subject #5 at the location (3,3)

The template matching algorithm presented in [41–45] was applied to the HF-SCG signals. First, the normalized cross-correlation (NCC) function was computed between a heartbeat template, selected from a HF-SCG signal, and the whole signal. Particularly, a reference heartbeat was selected for each subject, by considering a time interval comprising both systolic and diastolic complexes, as described in [41–45], in all HF-FCG signals of the 4-by-4 accelerometer matrix. The signal patterns corresponding to the reference heartbeat were extracted from each of the 16 HF-SCG channels and used as templates for the computation of the NCC. Then, the NCC peaks corresponding to heartbeats were localized. Finally, as in [15], to obtain information on the morphology variation, a continuous time trend, namely the morphological similarity index (MSi), was obtained via spline interpolation of the NCC peaks using the MATLAB® function “*interp1*”. The MSi signal was further low-pass filtered via a zero-lag 4th order Butterworth filter with 0.5 Hz cut-off frequency. In the HF-SCG signals of the same subject, the heartbeat templates were chosen at the same point of the respiratory cycle. An excerpt of the NCC signal and the MSi obtained from subject #5 at the location (3,3) is depicted in Fig. 3.

### Inter-breath intervals estimation

**Fig. 4:**
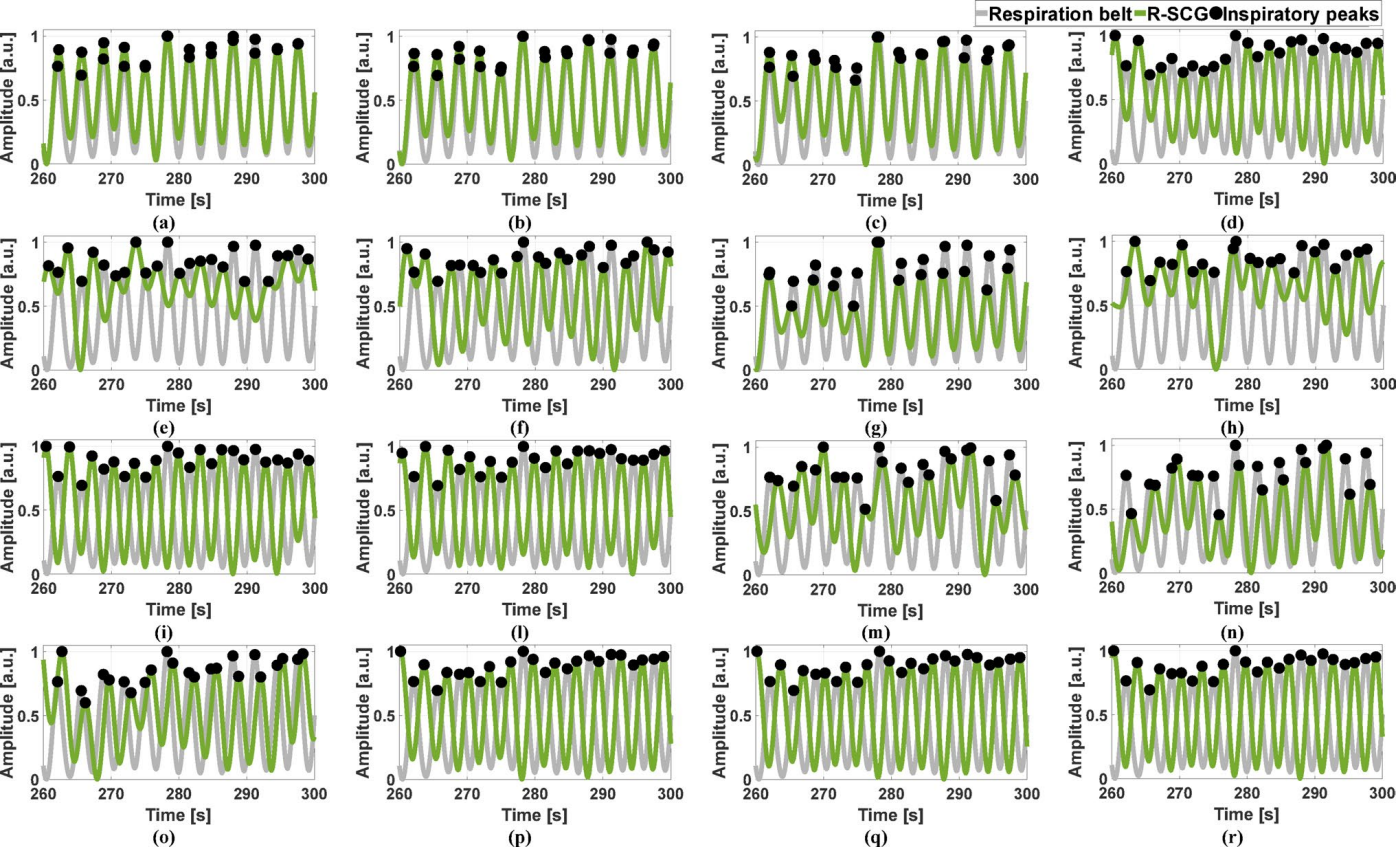
Some excerpts of the respiration belt signal (grey line) and the R-SCG signal (green line) from subject #2 for the 16 locations of the 4-by-4 accelerometer matrix. Black points mark the locations of the inspiratory peaks

**Fig. 5:**
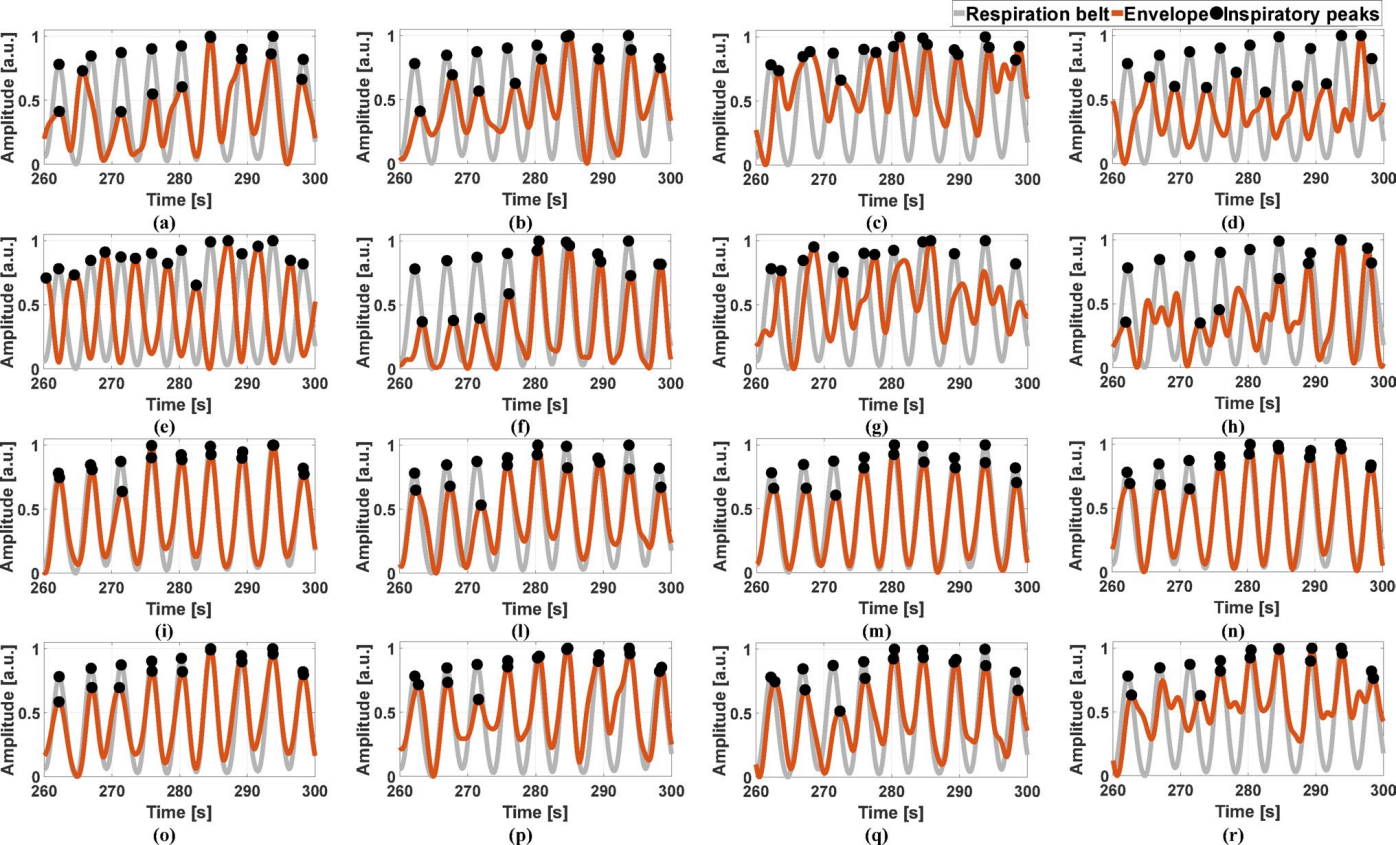
Some excerpts of the respiration belt signal (grey line) and the envelope of the HF-SCG signal (red line) from subject #4 for the 16 locations of the 4-by-4 accelerometer matrix. Black points mark the locations of the inspiratory peaks

**Fig. 6:**
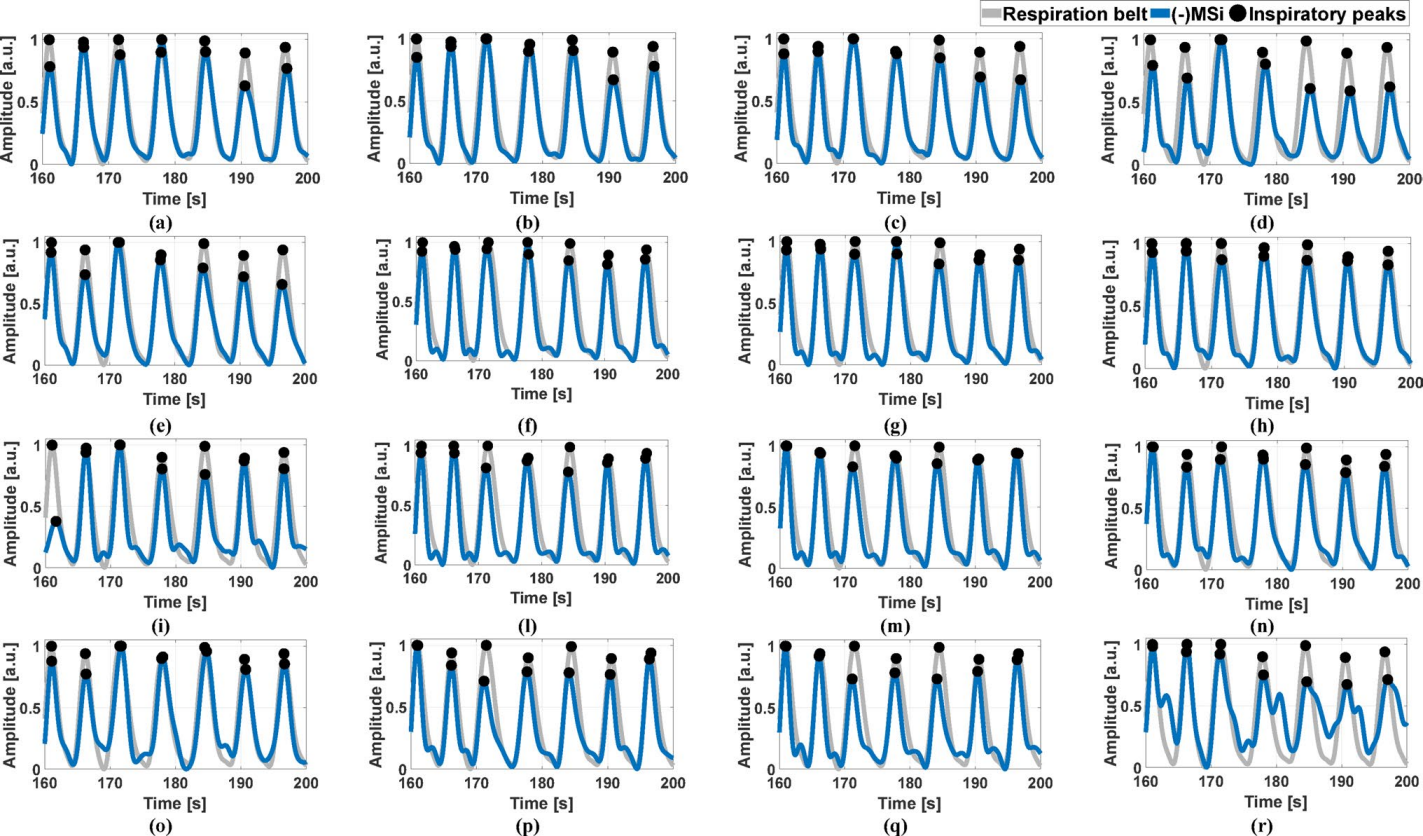
Some excerpts of the respiration belt signal (grey line) and the -MSi signal (blue line) from subject #5 for the 16 locations of the 4-by-4 accelerometer matrix. Black points mark the locations of the inspiratory peaks

To analyze the consistency within the respiratory cycle, the respiratory acts were identified in the reference signal, i.e., the respiration belt signal, and in the R-SCG signals, the envelopes of the HF-SCG signals, and the MSi signals by locating the positive inspiratory peaks via the MATLAB® function “*findpeaks*”. The number of true positives (TPs), false positives (FPs) and false negatives (FNs) were annotated for the three kinds of respiratory signals with respect to the reference signal. Particularly, TPs were considered as peaks that matched reference respiratory acts, while peaks that did not match any reference respiratory act were annotated as FPs, and missing peaks corresponding to reference respiratory acts were annotated as FNs. Figures 4, 5, and 6 show the respiratory acts detected in the R-SCG signals, the envelopes of the HF-SCG signals, and the MSi signals acquired from subjects #2, 4, and 5, respectively, at each location of the 4-by-4 accelerometer matrix, along with those identified in the reference respiration belt signals. These respiratory acts were deprived of false and missed detections. Finally, IBI estimates were obtained from the four signals as temporal differences between their consecutive inspiratory peaks.

### Statistical analyses

The sensitivity and positive predictive value (PPV) of respiratory acts detection were considered as performance evaluation metrics [46]. Furthermore, the IBI estimates obtained from the R-SCG signals, the envelopes of the HF-SCG signals, and the MSi signals were compared with those provided by the reference respiration belt signal via Passing-Bablok linear regression [47], correlation, and Bland–Altman [48] analyses. The IBI estimates corrupted by FPs and FNs were excluded from the analyses.

## Results

### Performance of respiratory acts detection

**Fig. 7:**
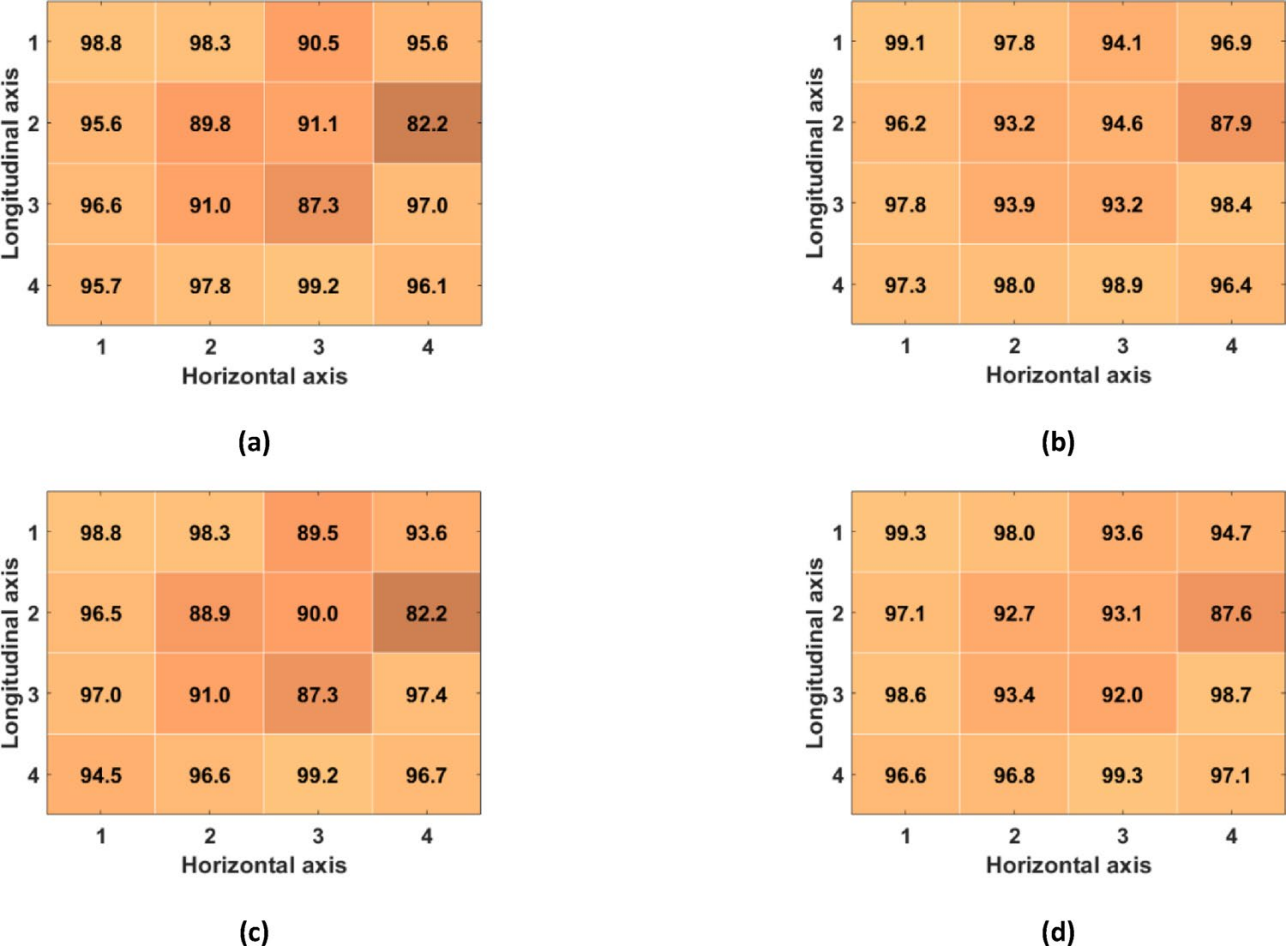
Performance of respiratory acts detection achieved across all subjects for the R-SCG signals for each location of the 4-by-4 accelerometer matrix: **a** macro-averaged sensitivity; **b** micro-averaged sensitivity; **c** macro-averaged PPV; **d** micro-averaged PPV

**Fig. 8:**
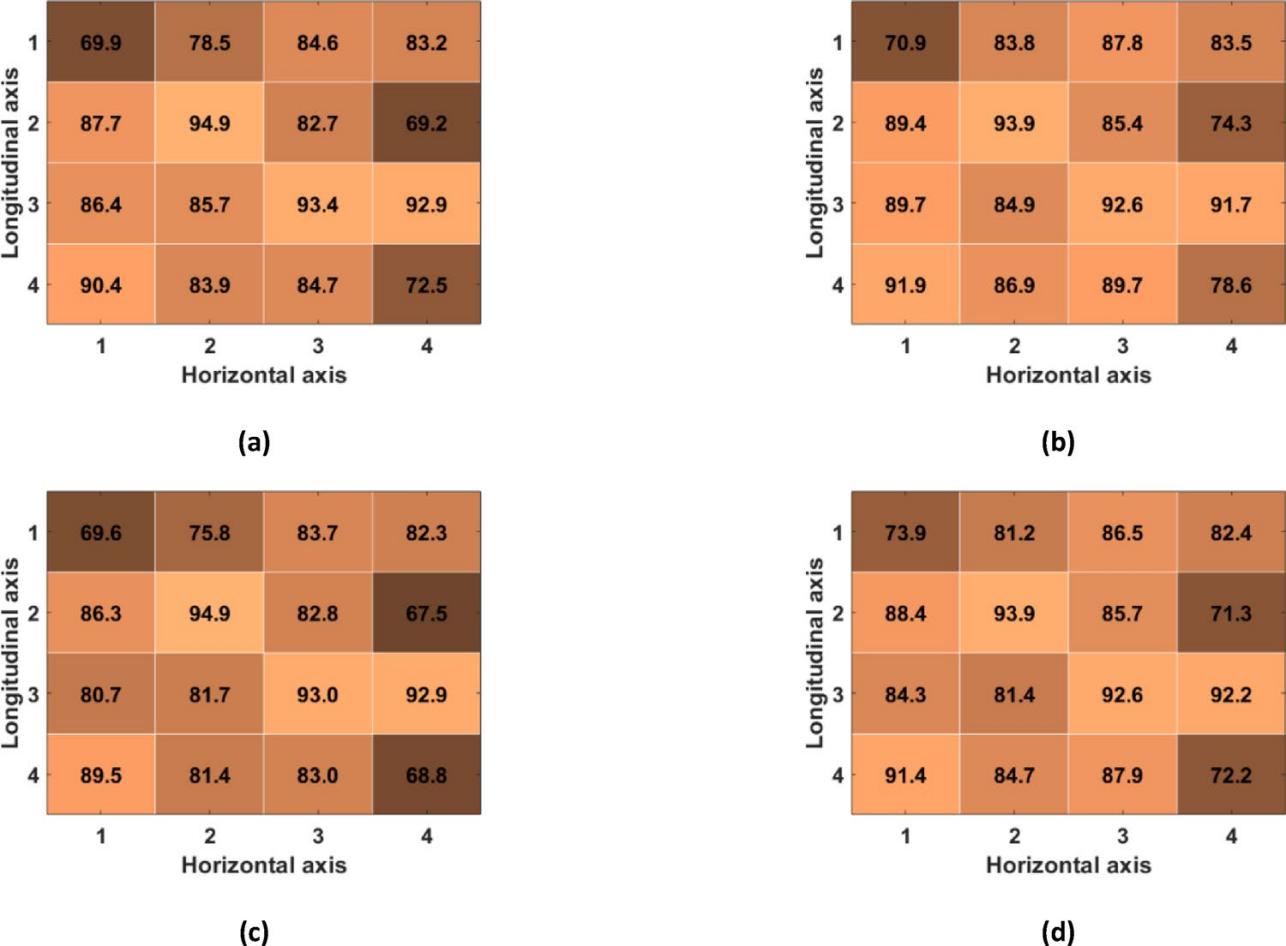
Performance of respiratory acts detection achieved across all subjects for the envelopes of the HF-SCG signals for each location of the 4-by-4 accelerometer matrix: **a** macro-averaged sensitivity; **b** micro-averaged sensitivity; **c** macro-averaged PPV; **d** micro-averaged PPV

**Fig. 9:**
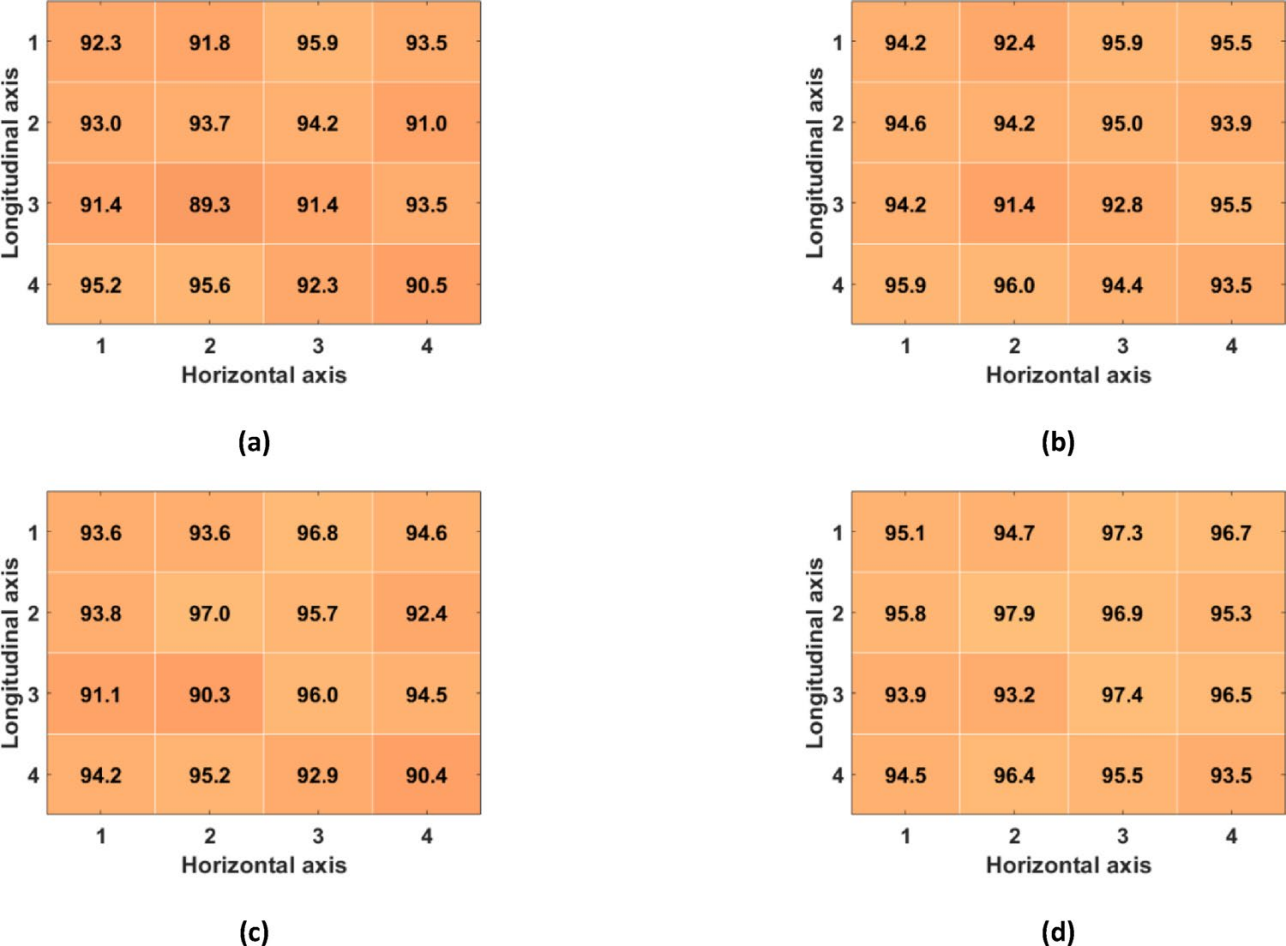
Performance of respiratory acts detection achieved across all subjects for the MSi signals for each location of the 4-by-4 accelerometer matrix: **a** macro-averaged sensitivity; **b** micro-averaged sensitivity; **c** macro-averaged PPV; **d** micro-averaged PPV

Table 1 reports the number of TPs, FPs and FNs detected across all subjects in the R-SCG signals, the envelopes of the HF-SCG signals, and the MSi signals for the 16 locations of the 4-by-4 accelerometer matrix, together with the total number of IBIs estimated. Figures 7, 8, and 9 show the performance of respiratory acts detection in terms of macro-averaged and micro-averaged sensitivity and PPV [46] respectively for the R-SCG signals, the envelopes of the z-SCG signals, and the MSi signals. Specifically, in R-SCG signals, values in excess of 90% were obtained for both macro-averaged sensitivity (except for the locations (2,2), (2,4), (3,2)), and PPV (except for the locations (1,3), (2,2), (2,4), (3,3)), while the micro-averaged sensitivity and PPV resulted in excess of 93% and 92%, respectively, except for the location (2,4). The envelopes of HF-SCG signals obtained macro-averaged sensitivity and PPV in excess of 82% and 80%, respectively, except for the locations (1,1), (1,2), (2,4), (4,4); while the micro-averaged sensitivity and PPV resulted in excess of 83% and 81%, respectively, except for the locations (1,1), (2,4) and (4,4). In the MSi signals, macro-averaged sensitivity and PPV in excess of 90% were obtained for each location, while the micro-averaged sensitivity and PPV resulted in excess of 91% and 93%, respectively.

**Table 1:** The number of TPs, FPs, and FNs detected across all subjects in the R-SCG signals, the envelopes of the HF-SCG signals, and the MSi signals, along with the number of IBIs

Loc	R-SCG				Envelope				MSi			
	TP	FP	FN	IBI	TP	FP	FN	IBI	TP	FP	FN	IBI
(1,1)	551	4	5	538	437	154	179	383	524	27	32	491
(1,2)	544	11	12	526	466	108	90	412	514	29	42	481
(1,3)	523	36	33	497	488	76	68	440	532	15	23	509
(1,4)	539	30	17	522	464	99	92	407	531	18	25	507
(2,1)	535	16	21	513	497	65	69	442	526	23	30	495
(2,2)	518	41	38	490	522	34	34	486	524	11	32	503
(2,3)	526	39	30	497	475	79	81	422	528	17	28	504
(2,4)	489	69	67	456	413	166	143	347	522	26	34	496
(3,1)	544	8	12	529	499	93	57	454	524	34	32	496
(3,2)	522	37	34	503	472	108	84	417	508	37	48	474
(3,3)	518	45	38	496	515	41	41	478	516	14	40	493
(3,4)	547	7	9	534	510	43	46	468	531	19	25	506
(4,1)	541	19	15	524	511	48	45	470	533	31	23	504
(4,2)	545	18	11	530	483	87	73	436	534	20	22	508
(4,3)	550	4	6	538	499	69	57	460	525	25	31	494
(4,4)	536	16	20	513	437	168	119	370	520	36	36	488

### Statistical analyses on IBIs measurements

Table 2, 3, and 4 summarize the results of correlation, Passing-Bablok linear regression, and Bland–Altman analyses performed on the IBIs estimated from, respectively, the R-SCG signals, the envelopes of the HF-SCG signals, and the MSi signals of all subjects. Particularly, almost unitary slopes and null intercepts were found for each location of the 4-by-4 accelerometer matrix for all respiratory signals. Moreover, in the R-SCG signals, the envelopes of the HF-SCG signals, and the MSi signals, Pearson’s correlation coefficients were found to be, respectively, in excess of 0.91 (except for the location (2,4)), 0.81, and 0.94 for each location. The Bland–Altman analysis reported non-significant biases, with zero comprised within their 95% confidence intervals for all respiratory signals, and limits of agreement (LoA) of at most [− 0.976; 1.08] s for the R-SCG signals, [− 1.33; 1.30] s for the envelopes of the HF-SCG signals, and [− 0.847; 0.945] s for the MSi signals.

**Table 2:** Results of statistical analyses performed on IBIs estimated from the R-SCG signals of all subjects

Loc	r	CI_r_	Slope	CI_slope_	Intercept (ms)	CI_Intercept_ (ms)	Bias (ms)	CI_bias_ (ms)	LoA (ms)	CI_LoAmin_ (ms)	CI_LoAmax_ (ms)
(1,1)	0.991	[0.990; 0.993]	1.00	[0.994; 1.01]	− 13.6	[− 49.5; 21.7]	− 1.0	[− 9.0; 10]	[− 289; 293]	[− 353; − 230]	[254; 476]
(1,2)	0.978	[0.974; 0.982]	1.01	[0.998; 1.02]	− 39.6	[− 87.9; 7.3]	− 2.5	[− 16.0; 10.4]	[− 510; 607]	[− 661; − 404]	[498; 777]
(1,3)	0.948	[0.938; 0.956]	1.01	[0.994; 1.03]	− 53.8	[− 126; 14.2]	− 9.0	[− 25.0; 16.6]	[− 768; 940]	[− 1070; − 608]	[668; 1169]
(1,4)	0.961	[0.953; 0.967]	1.01	[0.992; 1.03]	− 33.3	[− 107; 37.4]	7.5	[− 17.5; 21.0]	[− 739; 774]	[− 982; − 518]	[626; 1068]
(2,1)	0.969	[0.963; 0.974]	1.01	[0.995; 1.03]	− 46.3	[− 112; 17.9]	− 8.0	[− 27.0; 15.0]	[− 606; 721]	[− 850; − 516]	[594; 932]
(2,2)	0.950	[0.940; 0.958]	1.02	[1.00; 1.05]	− 81.8	[− 173; − 9.8]	− 7.5	[− 25.0; 17.3]	[− 731; 714]	[− 915; − 627]	[643; 940]
(2,3)	0.947	[0.937; 0.955]	1.03	[1.01; 1.06]	− 124	[− 215; − 36.7]	− 1.0	[− 18.0; 27.2]	[− 734; 754]	[− 919; − 520]	[578; 1214]
(2,4)	0.885	[0.863; 0.903]	1.07	[1.03; 1.12]	− 242	[− 383; − 103]	11.0	[− 5.0; 26.0]	[− 960; 1056]	[− 1365; − 750]	[764; 1336]
(3,1)	0.950	[0.941; 0.958]	1.00	[0.974; 1.02]	1.0	[− 85.8; 94.0]	1.0	[− 24.0; 20.0]	[− 721; 750]	[− 902; − 538]	[627; 1127]
(3,2)	0.914	[0.898; 0.927]	1.01	[0.975; 1.04]	− 30.9	[− 160; 81.0]	− 4.0	[− 26.0; 14.0]	[− 976; 1081]	[− 1241; − 835]	[888; 1308]
(3,3)	0.938	[0.926; 0.948]	1.02	[1.00; 1.04]	− 73.2	[− 142; − 2.9]	7.0	[− 3.0; 14.5]	[− 842; 794]	[− 1114; − 723]	[705; 1083]
(3,4)	0.979	[0.975; 0.982]	1.01	[1.00; 1.02]	− 35.9	[− 70.8; − 6.4]	2.5	[− 3.0; 8.5]	[− 512; 503]	[− 697; − 439]	[381; 657]
(4,1)	0.956	[0.947; 0.962]	1.00	[0.980; 1.02]	1.5	[− 72.1; 72.0]	1.5	[− 15.0; 14.0]	[− 713; 725]	[− 916; − 539]	[585; 944]
(4,2)	0.967	[0.961; 0.972]	1.01	[0.987; 1.03]	− 18.5	[− 90.2; 53.5]	8.0	[− 7.0; 17.0]	[− 593; 725]	[− 923; − 481]	[576; 950]
(4,3)	0.953	[0.945; 0.960]	1.02	[0.994; 1.05]	− 68.0	[− 162; 22.8]	0.5	[− 15.0; 16.0]	[− 845; 883]	[− 970; − 721]	[777; 1024]
(4,4)	0.962	[0.955; 0.968]	1.00	[0.984; 1.03]	− 17.6	[− 94.2; 55.9]	0.0	[− 13.0; 15.0]	[− 720; 732]	[− 910; − 626]	[632; 950]

**Table 3:** Results of statistical analyses performed on IBIs estimated from the envelopes of the HF-SCG signals of all subjects

Loc	r	CI_r_	Slope	CI_slope_	Intercept (ms)	CI_Intercept_ (ms)	Bias (ms)	CI_bias_ (ms)	LoA (ms)	CI_LoAmin_ (ms)	CI_LoAmax_ (ms)
(1,1)	0.840	[0.808; 0.867]	1.20	[1.12; 1.27]	− 710	[− 964; − 461]	− 18.0	[− 81.0; 21.0]	[− 1174; 1029]	[− 1532; − 924]	[866; 1428]
(1,2)	0.873	[0.848; 0.894]	1.17	[1.10; 1.23]	− 579	[− 815; − 342]	4.0	[− 29.4; 40.5]	[− 966; 878]	[− 1415; − 780]	[798; 1180]
(1,3)	0.907	[0.888; 0.922]	1.14	[1.09; 1.19]	− 517	[− 699; − 331]	0.0	[− 49.0; 37.8]	[− 1003; 1067]	[− 1329; − 798]	[930; 1451]
(1,4)	0.927	[0.912; 0.940]	1.09	[1.05; 1.13]	− 360	[− 533; − 217]	− 19.0	[− 51.0; 23.0]	[− 1078; 924]	[− 1281; − 961]	[849; 1225]
(2,1)	0.931	[0.918; 0.943]	1.08	[1.04; 1.12]	− 332	[− 495; − 176]	− 9.5	[− 54.5; 24.3]	[− 1069; 1102]	[− 1284; − 945]	[955; 1244]
(2,2)	0.932	[0.919; 0.942]	1.07	[1.04; 1.11]	− 312	[− 454; − 168]	− 10.0	[− 34.5; 28.0]	[− 1054; 1275]	[− 1128; − 858]	[1133; 1484]
(2,3)	0.926	[0.911; 0.939]	1.06	[1.02; 1.10]	− 257	[− 403; − 111]	− 19.0	[− 61.0; 22.0]	[− 997; 1298]	[− 1340; − 830]	[1043; 1495]
(2,4)	0.839	[0.804; 0.867]	1.23	[1.15; 1.33]	− 877	[− 1228; − 580]	− 40.0	[− 95.0; 18.0]	[− 1326; 1151]	[− 1400; − 1088]	[1010; 1524]
(3,1)	0.881	[0.859; 0.900]	1.14	[1.09; 1.20]	− 573	[− 780; − 376]	− 25.0	[− 61.0; 19.0]	[− 1138; 1120]	[− 1371; − 922]	[969; 1438]
(3,2)	0.925	[0.910; 0.938]	1.07	[1.03; 1.11]	− 256	[− 422; − 105]	8.0	[− 36.0; 47.0]	[− 962; 1031]	[− 1111; − 746]	[878; 1280]
(3,3)	0.953	[0.945; 0.961]	1.04	[1.01; 1.07]	− 147	[− 269; − 27.1]	8.5	[− 16.0; 24.5]	[− 797; 924]	[− 1134; − 646]	[830; 1082]
(3,4)	0.952	[0.942; 0.960]	1.05	[1.02; 1.07]	− 190	[− 295; − 97.3]	− 1.5	[− 24.0; 21.5]	[− 864; 825]	[− 1008; − 697]	[719; 1341]
(4,1)	0.938	[0.926; 0.948]	1.08	[1.04; 1.11]	− 324	[− 462; − 191]	− 14.0	[− 39.5; 29.2]	[− 847; 768]	[− 1137; − 749]	[682; 967]
(4,2)	0.897	[0.877; 0.914]	1.08	[1.04; 1.14]	− 324	[− 521; − 151]	6.0	[− 32.9; 55.0]	[− 1090; 1109]	[− 1267; − 888]	[931; 1293]
(4,3)	0.900	[0.881; 0.916]	1.08	[1.04; 1.14]	− 352	[− 549; − 165]	− 14.0	[− 72.4; 20.4]	[− 1113; 1051]	[− 1411; − 996]	[939; 1171]
(4,4)	0.815	[0.778; 0.847]	1.15	[1.09; 1.22]	− 518	[− 747; − 316]	− 6.0	[− 45.8; 39.0]	[− 1232; 1095]	[− 1.49; − 1.07]	[910; 1787]

**Table 4:** Results of statistical analyses performed on IBIs estimated from the MSi signals of all subjects

Loc	r	CI_r_	Slope	CI_slope_	Intercept (ms)	CI_Intercept_ (ms)	Bias (ms)	CI_bias_ (ms)	LoA (ms)	CI_LoAmin_ (ms)	CI_LoAmax_ (ms)
(1,1)	0.959	[0.951; 0.966]	1.04	[1.02; 1.07]	− 181	[− 289; − 74.5]	− 1.0	[− 31.0; 21.0]	[− 847; 749]	[− 1021; − 725]	[639; 950]
(1,2)	0.955	[0.946; 0.962]	1.03	[1.01; 1.06]	− 158	[− 261; − 51.3]	− 5.0	[− 34.0; 18.0]	[− 704; 796]	[− 857; − 604]	[673; 1048]
(1,3)	0.957	[0.949; 0.964]	1.04	[1.01; 1.06]	− 164	[− 258; − 70.3]	− 14.0	[− 34.0; 5.0]	[− 793; 912]	[− 1033; − 665]	[738; 966]
(1,4)	0.967	[0.961; 0.972]	1.03	[1.01; 1.05]	− 132	[− 221; − 50.8]	− 12.0	[− 29.0; 13.0]	[− 591; 637]	[− 730; − 530]	[545; 800]
(2,1)	0.946	[0.936; 0.954]	1.06	[1.04; 1.09]	− 288	[− 409; − 176]	− 12.5	[− 47.5; 16.0]	[− 766; 762]	[− 852; − 646]	[715; 912]
(2,2)	0.966	[0.960; 0.971]	1.03	[1.01; 1.06]	− 136	[− 221; − 52.0]	5.0	[− 14.0; 25.0]	[− 727; 733]	[− 865; − 554]	[631; 823]
(2,3)	0.963	[0.956; 0.969]	1.02	[1.01; 1.05]	− 107	[− 183; − 29.6]	− 2.5	[− 25.5; 15.0]	[− 696; 709]	[− 1024; − 563]	[542; 992]
(2,4)	0.954	[0.946; 0.961]	1.02	[1.00; 1.05]	− 105	[− 191; − 27.7]	− 11.5	[− 33.0; 15.0]	[− 707; 945]	[− 933; − 550]	[711; 1135]
(3,1)	0.957	[0.949; 0.964]	1.03	[1.01; 1.05]	− 117	[− 195; − 44.6]	− 12.0	[− 27.0; 2.2]	[− 798; 787]	[− 928; − 602]	[608; 1030]
(3,2)	0.966	[0.960; 0.972]	1.02	[1.01; 1.05]	− 98.2	[− 183; − 17.4]	6.0	[− 22.0; 23.0]	[− 702; 566]	[− 807; − 462]	[510; 797]
(3,3)	0.965	[0.959; 0.971]	1.03	[1.01; 1.05]	− 127	[− 215; − 43.8]	− 7.5	[− 29.0; 9.0]	[− 691; 676]	[− 805; − 530]	[523; 797]
(3,4)	0.951	[0.941; 0.958]	1.05	[1.02; 1.07]	− 200	[− 305; − 99.7]	− 8.0	[− 32.0; 14.0]	[− 818; 837]	[− 924; − 664]	[624; 1096]
(4,1)	0.966	[0.960; 0.972]	1.03	[1.00; 1.05]	− 128	[− 226; − 31.7]	− 16.0	[− 42.0; 15.0]	[− 666; 720]	[− 855; − 591]	[592; 830]
(4,2)	0.953	[0.944; 0.960]	1.02	[0.997; 1.04]	− 73.6	[− 150; 7.1]	− 6.5	[− 31.5; 24.0]	[− 755; 753]	[− 951; − 587]	[549; 896]
(4,3)	0.959	[0.952; 0.966]	1.02	[1.00; 1.05]	− 87.6	[− 171; − 7.2]	5.5	[− 14.7; 28.0]	[− 628; 830]	[− 760; − 516]	[637; 1000]
(4,4)	0.952	[0.942; 0.959]	1.05	[1.02; 1.08]	− 177	[− 278; − 76.8]	5.5	[− 20.4; 24.0]	[− 797; 850]	[− 923; − 649]	[740; 1045]

## Discussion

Very accurate localization of respiratory acts and measurement of inter-breath intervals were possible in all 16 positions over the chest, thus confirming that sensor placement has no practical impact on respiratory signals based on thoracic movements. This may be explained by the fact that the accelerometer matrix is integral with the thorax during respiration; so, each accelerometer is highly sensitive to chest motion along the head-to-foot direction. While R-SCG provided direct measurements of respiratory chest movements, amplitude modulations and morphological changes of SCG signals provided two indirect measurements of respiratory activity based on cardiorespiratory interactions. Amplitude modulations of SCG signals can be mainly ascribed to two phenomena: (1) changes in mutual distances between the stationary accelerometers and the heart moving with the diaphragm, that modify the attenuation of cardiac vibrations that reach the chest surface; (2) changes in intrathoracic pressure, to which the heart responds with an increased contraction force that produces more powerful vibrations. In addition to modifying the lengths of the paths travelled by cardiac vibrations, changes in lungs volumes also modify the mechanical characteristics of the materials that are on those paths. It is reasonable to consider that this phenomenon alters the propagation of different components of cardiac vibrations, thus leading to changes in the overall morphology of SCG signals captured on chest surface. Moreover, the heart response to changes in intrathoracic pressure modifies pressure gradients across the heart valves, which may influence the timing and dynamics of their openings and closures, eventually causing morphological changes in cardiac vibrations as well. In this study, amplitude modulations and morphological changes were both clearly observed in SCG signals via a quantitative analysis of temporal trends of SCG envelope and morphological similarity index. These temporal trends showed close relationships with respiratory activity, to the point that almost all respiratory acts were precisely localized and provided accurate estimates of inter-breath intervals. Although some variations among sensor locations were observed for amplitude modulation of SCG signals, no general conclusions could be drawn from the analyzed data, because of the limited number of subjects involved. The novelty of this study is three-fold. It presented for the first time a continuous description of morphological changes in SCG signals. In fact, previous studies have highlighted at most the existence of only two different SCG morphologies corresponding to high and low lung volume conditions [18]. Instead, the results of this study showed that SCG signals exhibit several different morphologies that appear periodically almost at the same points within respiratory cycles. Moreover, this study presented a thorough evaluation of the relationship between respiration and the related alterations induced on SCG signals, which has not been carried out previously. Similar results have been reported by some of the authors in [14, 15] for HF-FCG signals, which confirm the very high similarity with SCG signals. Finally, this study also addressed for the first time the impact of SCG sensor placement on the extraction of respiratory information. Indeed, previous studies considered only SCG signals acquired at the xiphoid, the 4th intercostal space or the midclavicular region [9, 16–19, 34]. This study has some limitations, mainly due to the experimental conditions of the data analyzed. Specifically, only SCG recordings acquired from subjects at rest in supine position were considered, so the impact of body posture and movements could not be assessed. Moreover, SCG signals were recorded only during quite breathing, which prevented any investigation of cardiorespiratory interactions at different respiratory rates and depths via analysis of SCG recordings. Finally, this study considered only SCG signals from young healthy subjects. Cardiorespiratory interactions are known to be altered by aging [49] and cardio-pulmonary diseases [50–54], hence extending the analyses presented in this study to subjects with these health conditions could widen the scope of SCG to pervasive monitoring applications.

## Conclusions

This study investigated the impact of SCG sensor placement on respiration monitoring and evaluation of cardiorespiratory interactions. To this end, multichannel SCG recordings from a public repository [36] were analyzed, which had been acquired via a 4-by-4 matrix of triaxial accelerometers, firmly attached to subjects’ chest. SCG signals had been recorded from healthy subjects at rest in supine position during quiet breathing, simultaneously with a respiration belt signal, which monitored breathing activity. Different analyses were carried out in this study to evaluate the possibility of extracting respiratory information from accelerometric recordings, as well as to study the effect of cardiorespiratory interactions on SCG signals. In particular, direct measurements of respiratory chest movements were extracted from cranio-caudal accelerometric recordings by capturing their baseline wandering. In addition, two indirect measurements of respiratory activity were also obtained by considering amplitude modulations and morphological changes of dorso-ventral SCG recordings caused by cardiorespiratory interactions. This study presented, for the first time, a continuous description of morphological changes in SCG signals, a thorough evaluation of the relationship between respiration and the related alterations induced on SCG signals, and an assessment of the impact of sensor placement on the extraction of respiratory information from SCG. Very accurate localization of respiratory acts and measurement of inter-breath intervals were possible in all three kinds of respiratory signals analyzed. No particular impact of sensor location was observed in respiratory signals based on thoracic movements and morphological changes of SCG signals. Some differences were found for amplitude modulation of SCG signals acquired at different sensors locations, but no general conclusions could be drawn because of the limited number of subjects involved. Analyses of SCG signals from a larger cohort of subjects, including elderly people and patients with cardio-pulmonary diseases, are envisioned in the future, also considering different body postures and movements, as well as wider ranges of respiratory rates and depths.

## Data Availability

Data considered in this study is available in a public repository.
